# Dominant Eye and Visual Evoked Potential of Patients with Myopic Anisometropia

**DOI:** 10.1155/2016/5064892

**Published:** 2016-06-02

**Authors:** Qing Wang, Yili Wu, Wenwen Liu, Lin Gao

**Affiliations:** ^1^Department of Ophthalmology, The Affiliated Hospital of Qingdao University, Qingdao 266001, China; ^2^Department of Epidemiology and Health Statistics, The Medical College of Qingdao University, Qingdao 266001, China; ^3^The Medical College of Qingdao University, Qingdao 266001, China

## Abstract

A prospective nonrandomized controlled study was conducted to explore the association between ocular dominance and degree of myopia in patients with anisometropia and to investigate the character of visual evoked potential (VEP) in high anisometropias. 1771 young myopia cases including 790 anisometropias were recruited. We found no significant relation between ocular dominance and spherical equivalent (SE) refraction in all subjects. On average for subjects with anisometropia 1.0–1.75 D, there was no significant difference in SE power between dominant and nondominant eyes, while, in SE anisometropia ≥1.75 D group, the degree of myopia was significantly higher in nondominant eyes than in dominant eyes. The trend was more significant in SE anisometropia ≥2.5 D group. There was no significant difference in higher-order aberrations between dominant eye and nondominant eye either in the whole study candidates or in any anisometropia groups. In anisometropias >2.0 D, the N75 latency of nondominant eye was longer than that of dominant eye. Our results suggested that, with the increase of anisometropia, nondominant eye had a tendency of higher refraction and N75 wave latency of nondominant eye was longer than that of dominant eye in high anisometropias.

## 1. Introduction 

Ocular dominance is defined as the tendency to prefer visual input from one eye over that from the other both in fixation and in attention or in perceptive function [1, 2]. Anisometropia or a relative difference in the refractive state of the two eyes is common in myopic patients. It is hypothesized that ocular dominance could affect myopia, and the effect would be stronger on those with anisometropic myopia. Several studies have explored the correlation of eye dominance and ocular growth and refraction [3–8]. However, there were no consistent results. Ito et al. [3] and Linke et al. [4] found that nondominant eyes had a greater myopic refractive error and longer axial length compared to dominant eyes, while Cheng et al. [8] and most recently Jiang et al. [9] find that dominant eyes were more myopic in myopic anisometropic subjects. Other studies [5–7] found no apparent association between refraction and ocular dominance or no significant effect of ocular dominance on myopia development.

Ocular dominance plays an important role in reading [10]. Vincent et al. [11] found that the dominant eye is showing significantly greater accommodative response during binocular viewing. Based on the assumption that blur is easier to be suppressed in nondominant eye, dominant eye is usually corrected for distant vision and nondominant eye is usually corrected for near vision in monovision design. The success of monovision for presbyopic correction also demonstrates the impact of ocular dominance on visual outcomes [12].

Visual evoked potential (VEP) measures the electrical response of the primary visual cortex to visual stimuli. As a result of hand and cerebral hemisphere dominance, visual cortices tend to prefer visual input from the dominant eye over that from the nondominant eye [13]. Ocular dominance affects magnitude of dipole moment [14], which indicates that ocular dominance affects laterality in the activity of the primary visual cortex; thus the amplitude of VEP wave of dominant eye is larger than that of nondominant eye [15, 16].

However, the role of dominant eye in visual, refractive, and oculomotor processes remains obscure [17]. In this study, we analyzed the association between ocular dominance and refractive asymmetry in a large series of refractive surgery candidates. To determine the effect of eye dominance on VEP waves, we further examined P-VEP of 40 high myopic anisometropias (refraction difference between both eyes is larger than 2.0 D). The association between ocular dominance, VEP, and myopic anisometropia might help to elucidate the mechanisms underlying myopia occurrence and development.

## 2. Subjects and Methods

### 2.1. Subjects

In our study, we selected 1771 relatively young candidates aged from 18 to 42 years who performed refractive surgery (LASIK or LASEK) from January 2011 to October 2013 in our refractive clinics. Candidates who exceeded the range for laser vision correction were excluded. To avoid any effect of amblyopia on ocular dominance, spectacle-corrected visual acuity (logMAR) worse than 0.00 in each eye or history of amblyopia or strabismus was excluded. All cases had no history of refractive or other ocular surgery or clinically significant retinal pathology, glaucoma, or systemic diseases or any other diseases that probably affect the visual function. A detailed general ophthalmological preoperative examination was performed including uncorrected distance visual acuity (UDVA), corrected distance visual acuity (CDVA), manifest refraction, cycloplegic refraction, tonometry, pupillometry, cornea pachymetry, corneal topography (WaveLight Allegro Topolyzer, Erlangen, Germany, or Galilei, Switzerland), wave-front aberration (WaveLight Allegro Analyzer, Erlangen, Germany), slit lamp examination, and funduscopy. All refractive data were converted into minus cylinder form to prevent confusion during data analysis. Verbal and written consent were obtained from all participants. The study was conducted in adherence to the tenets of the Declaration of Helsinki and approved by the ethics committee of Qingdao University.

### 2.2. Methods

To determine ocular dominance or motor dominance, the hole-in-the-card test [18] (Dolman method) was performed. The patient holds a card with a hole in the middle using both hands and is asked to view (manifest correction) a 6-m distant target through the hole. The observer then occludes each eye alternately to establish which eye is aligned with the hole and the target. The aligned eye is considered to be the dominant eye. Then, the subject moves the card slowly toward his/her face without losing alignment with the fixation point until the hole is over an eye, and the eye is considered to be the dominant one. If we did not observe a clear preference, ocular dominance was classified as undetermined.

The higher-order root-mean-square (RMS) wave-front aberrations at 5-mm zone were measured by the WaveLight Allegro Analyzer (Erlangen, Germany). The measurement was performed three times at least, and the mean of three readings was collected.

Monocular P-VEP test was examined by LKC'S UTAS visual electrodiagnostic test system, and the recording of VEPs was in low photopic lighting conditions (illuminance at cornea was 5 lux) in a sound-attenuated room. Average pupil diameter was 5.7 ± 0.4 mm. VEPs were elicited using reversing 12 arcmin (3 cpd) checks at a rate of 4 reversals/s (2 Hz) with square wave modulation. The stimulus subtended a circular field of 7° with 100% contrast and a constant mean luminance of 30 cd/m^2^. The circular field was surrounded by a background of the same mean luminance and color (illuminant C, chromatic coordinates: *x* = 0.310 and *y* = 0.316). The patient was seated comfortably and located 1 meter away from the screen. Fixation was achieved by encouraging the subject to relax and to fixate on the central fixation light. Each VEP trace was the average of 64 epochs of 1-second duration each, as suggested by the International Society of Clinical Electrophysiology of Vision (ISCEV). According to ISCEV guidelines, before signal averaging, computerized artifact rejection was performed to discard epochs in which eye position deviated or blink or amplifier blocking occurred.

The VEP examination was repeated for three times for each eye. N75 latency and P100 amplitude and latency scores were calculated on the average waveform. It required manual definition of the lowest negative peak (N75) before P100 peak. Amplitude was scored as the difference between these two points and latency was scored as the time difference between N75 lowest peak or P100 peak and stimulus onset.

### 2.3. Statistical Analysis

The difference in refractive parameters (SE, astigmatism) and wave-front aberrations and N75 and P100 amplitude and latency between dominant and nondominant eyes was compared with paired Student's *t*-test and Wilcoxon signed rank test. The difference in eye dominance between the males and the females was examined by chi-square test. *P* < 0.05 was defined as statistically significant. To determine the relationship between degree of myopia and N75 latency, graphs were plotted with the degree of myopia on the *x*-axis versus the amount of N75 latency on the *y*-axis. In order to observe subjects who had obvious anisometropia ≥1.0 D, anisometropia was further divided into three SE subgroups (1–1.74, 1.75–2.49, and ≥2.5 D) to confirm whether the dominant eye had a higher degree of myopia.

## 3. Results

A total of 1771 eligible subjects (1161 males, 610 females) were enrolled. The mean age was 22.10 ± 4.58 years (18–42 years). The mean spherical equivalent (SE) refraction was −5.19 ± 2.13 D, and there was no significant difference in SE between the right eye (−5.3 ± 2.11 D) and the left eye (−5.08 ± 2.14 D; *P* = 0.453). Ocular dominance of right eye and left eye was 62.2% (*n* = 1102) and 36.1% (*n* = 638), respectively. And 1.75% (*n* = 31) of subjects had no obvious eye dominance. There were 325 subjects who had anisometropia SE ≥1.00 D (ranged from 1 to 5.75 D). 40 subjects with anisometropia SE ≥2.0 D were further examined with VEP. The average age of those 40 subjects (28 males, 12 females) was 28.11 ± 4.56 years (18–34 years old). Average SE was −4.97 ± 2.53 D (range from −0.5 to −12.00 D).

### 3.1. Ocular Dominance and Sex

65.5% of the subjects were males. The mean age of the male group was 20.9 ± 3.5 years, mean SE was −4.79 ± 1.99 D (range between −15.75 and 0.25 D), and mean astigmatism was −0.6 ± 0.6 D. For the female subjects, mean age was 24.4 ± 5.4 years, mean SE was −5.96 ± 2.17 D (range between −14.75 and 0.25 D), and mean astigmatism was −0.5 ± 0.6 D. There was no difference in eye dominance between the males and the females (*x* ^2^ = 0.274, *P* = 0.601). For most subjects (both males and females), dominant eye was the right eye (Table 1).

### 3.2. Ocular Dominance and SE in Anisometropia

In the whole study population, there was no significant difference (*P* = 0.353) in the amount of myopia between the dominant eye (−5.14 ± 2.15 D) and the nondominant eye (−5.23 ± 2.26 D). For subjects with anisometropia 1.0–1.75 D, there was no significant difference in SE power (*n* = 202; *P* = 0.348) between dominant and nondominant eyes. In subjects with SE anisometropia 1.75–2.5 D, the degree of myopia was significantly higher (*P* = 0.008) in nondominant eyes (−5.8 ± 2.8 D) than in dominant eyes (−5.2 ± 2.5 D). The trend of the nondominant eye to be more myopic was more significant (*P* = 0.002) for SE anisometropia ≥2.5 D (Table 2).

### 3.3. Ocular Dominance and Astigmatism in Anisometropias

In the whole study population, the astigmatism in dominant eyes was −0.50 ± 0.58 D and in nondominant eyes was −0.52 ± 0.61 D. In 790 (44.6%) anisometropia subjects (anisometropia ≥0.50 D), there was also no significant difference in astigmatism between dominant and nondominant eyes. For subjects with anisometropia 1–1.74 D, the amount of astigmatism was −0.47 ± 0.63 D in dominant eyes and −0.41 ± 0.57 D in nondominant eyes. With the increase of anisometropia, astigmatism is also increased (Table 3). There was no significant difference in astigmatism between dominant and nondominant eyes in any anisometropia groups.

### 3.4. Ocular Dominant and Wave-Front Aberration in Anisometropias

In the whole study population, there was no significant difference (*P* = 0.241) in wave-front aberration between the dominant eye (0.23 ± 0.13) and the nondominant eye (−0.24 ± 0.14). As shown in Table 4, with the increase of anisometropia, the aberration increased and the nondominant eye appeared to be with higher aberration compared to the dominant eye, but the difference was not significant.

### 3.5. VEP Results in Selected Anisometropia Subjects

For the 40 subjects with anisometropia more than 2 D, nondominant eyes have higher myopia SE than dominant eyes (Table 5). The difference of astigmatism between dominant eyes and nondominant eyes was not significant (*P* = 0.601), with the same results for the axis of astigmatism and wave-front aberration.

**Figure 1:**
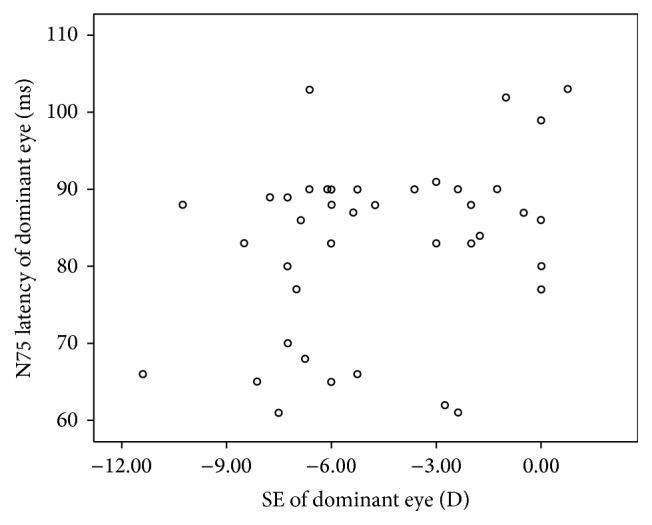
Scatter spots assessing correlation between spherical equivalent (SE) and N75 latency in dominant eye. In dominant eye, the SE was not correlated with N75 latency, but the *P* value was very close to 0.05, *r* = 0.310 and *P* = 0.052 (Pearson correlation).

The N75 latency of dominant eyes (83.0 ± 11.5 ms) was shorter than that of nondominant eyes (89.4 ± 11.6 ms) in the selected anisometropias (*Z* = −2.884, *P* = 0.004). However, the P100 latency between the dominant and nondominant eyes was not significantly different (*Z* = −0.325, *P* = 0.745). The correlation between SE and N75 latency was not significant, but *P* value was very close to 0.05 (*P* = 0.052, Figure 1). The wave-front aberration had no correlation with N75 latency both in dominant eyes and in nondominant eyes as shown in Table 5.

## 4. Discussion

There are several reports about ocular dominance and myopia and also several researches about VEP and refraction. However, there are few investigations about ocular dominance and VEP results in myopia anisometropia. In our study, we investigated the association between ocular dominance and VEP and myopic anisometropia, which might help to elucidate the mechanisms underlying myopia occurrence and development.

Ocular dominance could be classified into sighting, motor, and sensory dominance. Sighting dominance [1, 2] refers to the preferential use of one eye over the fellow eye in fixating a target. Most previous studies used the hole-in-the-card test to measure sighting dominance [3–8]. Sensory dominance occurs when the perception of a stimulus to one eye dominates the other in retinal rivalry conditions [19]. It can be attributed to an interocular imbalance of the underlying inhibitory neural mechanism. By examining sensory eye dominance, Jiang et al. [9] found that the dominant eyes were more myopic in myopia anisometropic subjects and less hyperopic in hyperopic anisometropic subjects and concluded that degree of ocular sensory dominance is associated with interocular refractive error difference.

Using the hole-in-the-card test, we found that right eye ocular dominance was 62.2% and left eye dominance was 36%. 1.75% subjects had no obvious eye dominance. The rate of right eye dominance in males (62.9%) was similar to that in females (64.2%). These results were similar to previous studies [4, 5, 8], and no difference was found in mean SE between both eyes.

Ocular dominance was thought to be independent of refraction [20]. However, Cheng et al. [8] showed that dominant eyes had a significantly greater myopic SE than nondominant eyes in adult subjects; several other institutions had performed similar investigation but did not find consistent results. A research in Singapore children found that ocular laterality and dominance had no significant effect on spherical equivalence [7]. Another 2-year longitudinal study also found that ocular dominance had no significant effect on the myopia development [6]. Most recently, the largest study conducted by Linke et al. [4] which consisted of 9983 individuals was to find out if ocular dominance has a role in the progression of myopia. Converse to Cheng's results, the study found that the nondominant eye usually is more myopic (SE) in anisometropic subjects. This trend reached statistical significance for anisometropia >2.5 D (*n* = 278, *P* < 0.001). The study concluded that the higher the amount of SE anisometropia, the greater the likelihood that the nondominant eye was more myopic than the dominant eye. Our study which consisted of 1771 adults found that, in low anisometropia (1–1.75 D), dominant eyes (−5.55 ± 2.09 D) had no more myopia than nondominant eyes (−5.63 ± 2.39 D), which was similar to Cheng's study in anisometropia <1.75 D. However, in high anisometropias (≥1.75 D), our result was consistent with Linke's study [4], in that the nondominant eye (−5.79 ± 2.84 D) was more myopic than the dominant eye (−5.31 ± 2.49 D) in higher anisometropia (1.75–2.5 D, *P* = 0.016); the trend was more significant in anisometropia ≥2.5 D (*P* = 0.002). Chia et al. [7] and Linke et al. [4] showed that astigmatism was significantly lower in dominant eyes of anisometropic subjects. But, in our study, there was no significant difference in astigmatism in all anisometropias (≥1 D).

The difference results between Chia et al.'s [7], Linke et al.'s [4], and our study may be caused by the difference in age and sample size of the anisometropic subjects. Our subjects ages were from 18 to 34, with a mean of 22.1 years, while, in Chia et al.'s and Linke's study, the mean age was 30.3 ± 9.5 years and 34.94 ± 9.3 years (ranging from 18 to 68 years). There were 790 anisometropia subjects in our study, which was larger than Chia et al.'s but smaller than Linke's. Another important reason was that the anisometropia was differently divided. In our study, all subjects divided into three SE subgroups which were 1–1.74, 1.75–2.49, and ≥2.5 D, while, in Linke's study, the grading was ≤0.49, 0.5–1.74, 1.75–2.49, and ≥2.5 D, and in Chia et al.'s the grading were 0.5 to 1.75 and >1.75 D. We were more tending to observe subjects who had obvious anisometropia ≥1.0 D.

Until now, there have been no other investigations yet about the relationship between wave-front aberration and eye dominance. Tian et al. [21], Vincent et al. [22], and most recently Hartwig et al. [23, 24] found no significant interocular differences for higher-order aberrations in myopia anisometropias. In our results, we found no significant difference in high-order aberrations between dominant eyes and nondominant eyes either in the whole study group or in anisometropia groups. Paquin et al. [25] and Marcos [26] found that high-order aberrations associated with higher degrees of myopia; however, there was no significant correlation between the high-order aberrations and SE (*r* = 0.03, *P* = 0.085, and data was not provided) in our results.

Ocular dominance is related to some ocular mechanism and function, such as eye movement [27] and amblyopia [28], and is important in monovision decision [10, 12]. VEP is an effective means to study visual mechanisms and cortex electrical activity. Ocular dominance affects laterality in the activity of the primary visual cortex [29]. As a result of hand and cerebral hemisphere dominance, the visual cortices tend to prefer visual input from the dominant eye over that from the nondominant eye [12], which means that the wave amplitude between dominant and nondominant eye may be different.

The motor response reaction is triggered by a critical level of electrical activity in the visual pathway prior to the onset of advanced visual processing. So N latency may be more sensitive in determining the difference between dominant eye and nondominant eye. In our myopia anisometropias (*A* ≥ 2.0), N75 latency of dominant eyes (83.0 ± 11.5 ms) was shorter than that of nondominant eyes (89.4 ± 11.6 ms) (*n* = 40, *Z* = −2.884, and *P* = 0.004), while neither the p100 latency nor P100 amplitude showed difference between dominant and nondominant eyes. VEP had been shown to be affected by a number of influences in the normal adult healthy eye [29, 30]. The amplitude of VEP wave is greatest when the image is in focus and decreases as the image is defocused, which has been used as the basis for objective refraction of the eye [31]. However, with proper controls, the pattern VEP test can be used for objective assessment of visual function [30]. In our anisometropia subjects, all the eyes were corrected to 0.00 (logMAR) or better, leading to little change in p100 either in latency or in amplitude. Although there was no statistical significance for the correlation between SE and N75 in the current study, *P* value of 0.052 was very close to 0.05. One possible reason was that the correlation was too weak to test because of the small sample size. Our results suggested that refraction might also affect the N75 latency in dominant eye of high anisometropias. It was reported that cricketers had a faster N75 latency, but there was no correlation or difference between eye dominance and any characteristics of the VEP in their subjects [32]. Future studies should focus on the visual cortices electrophysiology in the development of visual imbalance between two eyes.

In summary, our study demonstrated a high rate of right eye dominance without gender deviation in a relatively young population. For all the candidates, there was no difference between dominant and nondominant eyes either in SE or in aberration except for astigmatism (our results showed a lower astigmatism in dominant eye). However, in low anisometropias, the dominant eye has a bit higher myopia than the nondominant eye, while, in mild and high anisometropias, the dominant eye was usually the lower myopia eye. In the selected high anisometropias, the dominant eye had a shorter N75 latency than the nondominant eye, which suggested that the delayed electrical activity in nondominant eye might play a role in the development of myopia.

There are several limitations in our study. The study was cross-sectional in nature and not longitudinal which limited the ability to attribute causation. Axial length was not measured, so the nature of the anisometropia was not clear whether it was refractive or axial. While this was a relatively large sample size of anisometropias, electrodiagnostics had only been examined in a smaller number of high anisometropias, which might induce a bias. A longitudinal study into the ocular changes of dominant and nondominant eyes during the development of anisometropia may provide further insight into the potential cause of this association.

## Figures and Tables

**Table 1:** Eye dominance between the males and the females (*n* = 1740).

	Left eye		Right eye		Total		*χ* ^2^	*P*
	*n*	%	*n*	%	*n*	%		
Male	423	37.1	717	62.9	1140	65.5	0.274	0.601
Female	215	35.8	385	64.2	600	34.5		

**Table 2:** Spherical equivalent (SE) of dominant eye and nondominant eye in different groups (Wilcoxon signed ranks; *n*, valid value, number of pairs).

Anisometropia	Dominant eye (D)	Nondominant eye (D)	*Z*	*P*
*A* ≥ 2.5 D (*n* = 40)	−4.95 ± 3.27	−7.07 ± 3.65	−3.047	0.002
1.75 D ≤ *A* < 2.5 D (*n* = 83)	−5.31 ± 2.49	−5.79 ± 2.84	−2.666	0.008
1 D ≤ *A* < 1.75 D (*n* = 202)	−5.55 ± 2.09	−5.63 ± 2.39	−0.939	0.094

**Table 3:** Astigmatism of dominant eye and nondominant eye in different groups (Wilcoxon signed ranks; *n*, valid value, number of pairs).

Anisometropia	Dominant eye (D)	Nondominant eye (D)	*Z*	*P*
*A* ≥ 2.5 D (*n* = 40)	−0.76 ± 1.11	−0.87 ± 0.83	−1.557	0.297
1.75 D ≤ *A* < 2.5 D (*n* = 83)	−0.70 ± 0.69	−0.74 ± 0.80	−0.331	0.688
1 D ≤ *A* < 1.75 D (*n* = 202)	−0.46 ± 0.58	−0.57 ± 0.54	−1.859	0.136

**Table 4:** Wave-front aberrations (RMS) of dominant eye and nondominant eye in different groups (Wilcoxon signed ranks; *n*, valid value, number of pairs).

Anisometropia	Dominant eye (*μ*m)	Nondominant eye (*μ*m)	*Z*	*P*
*A* ≥ 2.5 D (*n* = 40)	0.237 ± 0.088	0.261 ± 0.149	−0.660	0.543
1.75 D ≤ *A* < 2.5 D (*n* = 83)	0.259 ± 0.116	0.262 ± 0.333	−2.590	0.889
1 D ≤ *A* < 1.75 D (*n* = 202)	0.227 ± 0.075	0.236 ± 0.085	−2.176	0.660

**Table 5:** The visual evoked potential (VEP) results in selected anisometropia subjects *A* ≥ 2.0 (Wilcoxon signed ranks; *n*, valid value, number of pairs).

	Refraction (D)	Astigmatism (D)	Axis of astigmatism (°)	High-order aberration (*μ*m)	N75 latency (ms)	P100 latency (ms)	P100 amplitude (*μ*v)
Dominant eyes Nondominant eyes	−4.6 ± 3.2 −6.1 ± 3.2	−1.0 ± 1.0 −1.0 ± 1.0	48.3 ± 71.1 54.6 ± 75.7	0.3 ± 0.2 0.2 ± 0.1	83.0 ± 11.5 89.4 ± 11.6	119.4 ± 5.6 121.0 ± 12.7	9.02 ± 2.98 8.86 ± 2.85

Valid value *Z* *P* value	40 −3.298 0.001	40 −0.523 0.601	40 −0.543 0.609	35 −0.459 0.647	40 −2.884 0.004	40 −0.325 0.745	40 −0.357 0.547
